# Capsaicin Induces Autophagy and Apoptosis in Human Nasopharyngeal Carcinoma Cells by Downregulating the PI3K/AKT/mTOR Pathway

**DOI:** 10.3390/ijms18071343

**Published:** 2017-06-23

**Authors:** Yu-Tsai Lin, Hung-Chen Wang, Yi-Chiang Hsu, Chung-Lung Cho, Ming-Yu Yang, Chih-Yen Chien

**Affiliations:** 1 Department of Otolaryngology, Kaohsiung Chang Gung Memorial Hospital and Chang Gung University College of Medicine, Kaohsiung 83301, Taiwan; xeye@cgmh.org.tw; 2 Graduate Institute of Clinical Medical Sciences, College of Medicine, Chang Gung University, Taoyuan City 33302, Taiwan; 3 Kaohsiung Chang Gung Head and Neck Oncology Group, Cancer Center, Kaohsiung Chang Gung Memorial Hospital, Kaohsiung 83301, Taiwan; 4 Department of Neurosurgery, Kaohsiung Chang Gung Memorial Hospital, Kaohsiung 83301, Taiwan; m82whc@gmail.com; 5 Graduate Institute of Medical Science and Innovative Research Center of Medicine, College of Health Sciences, Chang Jung Christian University, Tainan 71101, Taiwan; jenway@mail.cjcu.edu.tw; 6 Department of Biological Sciences, National Sun Yat-sen University, Kaohsiung 80424, Taiwan; clcho@mail.nsysu.edu.tw

**Keywords:** capsaicin, autophagy, nasopharyngeal carcinoma, PI3K/AKT/mTOR pathway

## Abstract

Capsaicin is a potential chemotherapeutic agent for different human cancers. In Southeast China, nasopharyngeal carcinoma (NPC) has the highest incidence of all cancers, but final treatment outcomes are unsatisfactory. However, there is a lack of information regarding the anticancer activity of capsaicin in NPC cells, and its effects on the signaling transduction pathways related to apoptosis and autophagy remain unclear. In the present study, the precise mechanisms by which capsaicin exerts anti-proliferative effects, cell cycle arrest, autophagy and apoptosis were investigated in NPC-TW01 cells. Exposure to capsaicin inhibited cancer cell growth and increased G1 phase cell cycle arrest. Western blotting and quantitative real-time reverse transcription polymerase chain reaction (qRT-PCR) were used to measure capsaicin-induced autophagy via involvement of the class III PI3K/Beclin-1/Bcl-2 signaling pathway. Capsaicin induced autophagy by increasing levels of the autophagy markers LC3-II and Atg5, enhancing p62 and Fap-1 degradation and increasing caspase-3 activity to induce apoptosis, suggesting a correlation of blocking the PI3K/Akt/mTOR pathway with the above-mentioned anticancer activities. Taken together, these data confirm that capsaicin inhibited the growth of human NPC cells and induced autophagy, supporting its potential as a therapeutic agent for cancer.

## 1. Introduction

Nasopharyngeal carcinoma (NPC) is a malignant tumor of the head and neck. It is believed that Epstein-Barr virus, environmental carcinogens, ethnic background and dietary components all play an important role in the development of NPC [1]. Therefore, NPC displays a characteristic geographical distribution and is prevalent in Southeast Asia, the Mediterranean basin and the Middle East [2]. For example, it is estimated that the annual incidence of NPC varies from 15 to 50 cases per 100,000 individuals in South China [3]. Although advances in diagnostic imaging, radiation therapy and concurrent chemoradiotherapy have achieved better locoregional control, the final treatment outcomes are not satisfactory [4,5]. Therefore, the development of effective, but less toxic or natural extract-based therapies is necessary [6,7].

Capsaicin (8-methyl-*N*-vanillyl-6-nonenamide) is a naturally-occurring phytochemical and the major pungent constituent of hot chili peppers of the genus *Capsicum* (family Solanaceae), which are extensively used as food additives. The importance of capsaicin is confirmed by various existing pharmaceutical formulations and clinical applications [8]. Since the early 1980s, capsaicin has been used in clinics as a means of therapy to relieve pain [8]. Clinical studies demonstrate the efficacy of 8% patch capsaicin in the treatment of neuropathic pain to be similar to the efficacy of pregabalin [9,10]. Capsules containing doses within the range of 3.375–10 mg capsaicin have been shown to increase energy expenditure, fat oxidation and thermogenesis, but decrease appetite in humans [11]. Pharmaceutical formulations containing capsaicin have been used to treat nonallergic rhinitis and non-infectious perennial rhinitis [12,13]. Capsaicin has potential antitumor effects [14] and induces apoptosis in many types of malignant cell lines, including breast cancer [15,16], colon adenocarcinoma [17,18], esophagus epidermoid carcinoma [19], hepatocellular carcinoma [20,21], prostate cancer [22], head and neck cancer [23,24], and many others. The mechanism of capsaicin-induced apoptosis in cancer cells is not completely elucidated, but it involves increased intracellular Ca^2+^ levels [19,24], the generation of reactive oxygen species [19,21,24], disruption of mitochondrial membrane potential [17,24] and activation of transcription factors, such as STATs (signal transducer and activator of transcription protein family) [21]. Autophagy is a novel cancer therapy that could be an effective approach for alleviating treatment resistance in apoptosis-defective tumor cells [25]. It has been implicated in tumor growth and progression and explored as a potential therapeutic target [26,27]. Recently, a role for autophagy in capsaicin-induced cell death was proposed following reports indicating that capsaicin may induce autophagy, suggesting a promising therapeutic strategy for cancer [21,28,29,30]. However, only a few studies have examined capsaicin-induced apoptosis of NPC cells, and the effects of capsaicin on autophagic-associated pathways in NPC remain questionable.

Therefore, the current study investigated capsaicin-induced apoptosis and autophagy in NPC-TW01 cells. The results may expand our understanding of the apoptosis- and autophagy-relevant signaling pathways activated by capsaicin in cancer cells.

## 2. Results

### 2.1. Capsaicin Inhibits NPC-TW01 Cell Proliferation

**Figure 1:**
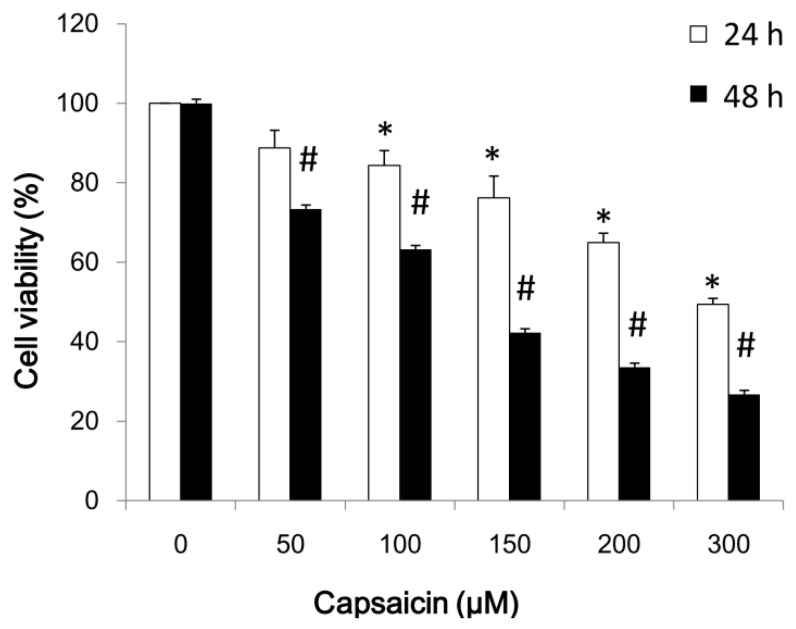
Capsaicin inhibits the viability of NPC-TW01 cells. Cell viability was determined by the MTT assay after treatment with various concentrations of capsaicin (0, 50, 100, 150, 200 and 300 µM) for 24–48 h. All data are expressed as a percentage of the control, which was considered to be 100%. The results are presented as the means ± SD and are representative of six independent experiments (*n* = 6). * *p* < 0.05 vs. control at 24 h. # *p* < 0.05 vs. control at 48 h.

The antitumor activity of capsaicin in NPC cells was investigated in vitro by treating NPC-TW01 cells with increasing doses of capsaicin (0, 50, 100, 150, 200 and 300 µM) for 24–48 h. The proliferation of capsaicin-treated cancer cells was then measured by the MTT assay (Figure 1). The findings indicated that the survival and proliferation of NPC-TW01 cells decreased with increasing concentrations of capsaicin. We also treated normal skin fibroblasts CCD966SK cells with capsaicin; no cytotoxicity was observed in the CCD966SK cells due to the capsaicin treatment (data not shown).

### 2.2. Capsaicin-Induced Cell Cycle Arrest in G1 Phase in NPC-TW01 Cells

**Figure 2:**
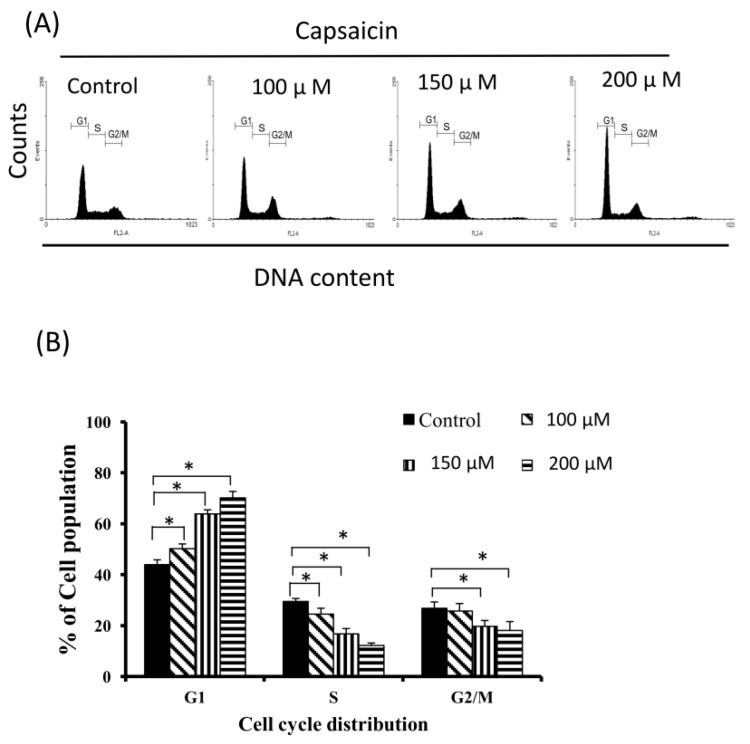

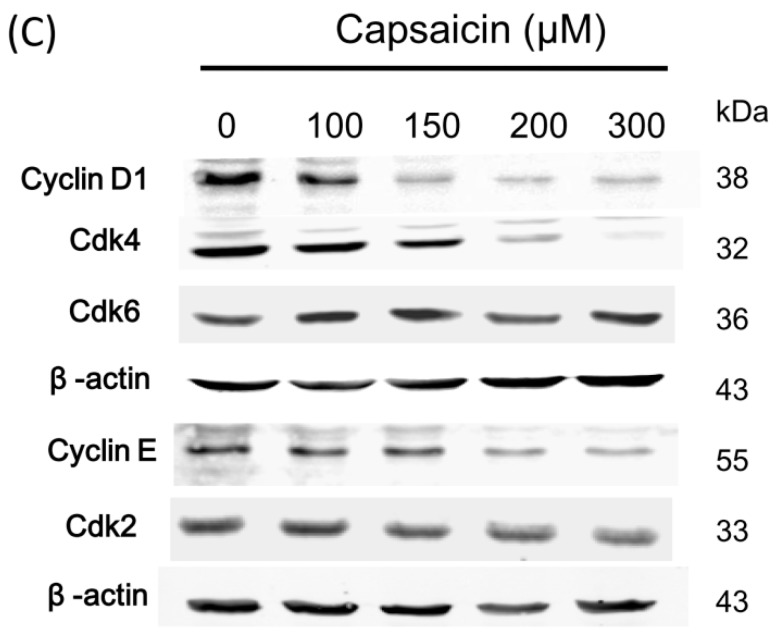
Capsaicin-induced accumulation of NPC-TW01 cells in G1 phase of the cell cycle. (**A**) Cell cycle distribution was analyzed by flow cytometry. NPC-TW01 cells were incubated with the indicated concentrations of capsaicin for 24 h, stained with PI and analyzed by flow cytometry. (**B**) The percentage of cells in the G1, S and G2/M phases. Values are reported as the means ± SD and are representative of three independent experiments (*n* = 3). * *p* < 0.05 vs. the respective controls. (**C**) Effects of capsaicin treatment on cell cycle-related proteins. NPC-TW01 cells were treated with capsaicin (0, 100, 150, 200 and 300 µM) for 24 h, and the levels of cyclin D1, CDK4, CDK6, cyclin E and CDK2 were determined by Western blotting.

The cell cycle distribution of capsaicin-treated cells was analyzed by flow cytometry. Cells were exposed to capsaicin for 24 h before processing and analysis. As shown in Figure 2A, capsaicin treatment increased the number of cells in G1 phase. Treatment with capsaicin also increased the number of cells in G0/G1 phase while simultaneously reducing the numbers of cells in S phase and G2/M phase (Figure 2B). Next, the effects of capsaicin treatment on the levels of cyclins and CDKs specific to G1-S-phase transition were assessed by Western blotting. Capsaicin caused a concentration-dependent decrease in cyclin D1, CDK4, cyclin E and CDK2 levels in NPC-TW01 cells, whereas CDK6 levels were not decreased significantly (Figure 2C).

### 2.3. Capsaicin Induces the Class III PI3K/Beclin-1/Bcl-2 Signaling Pathway to Contribute to Autophagy Activation in NPC-TW01 Cells

**Figure 3:**
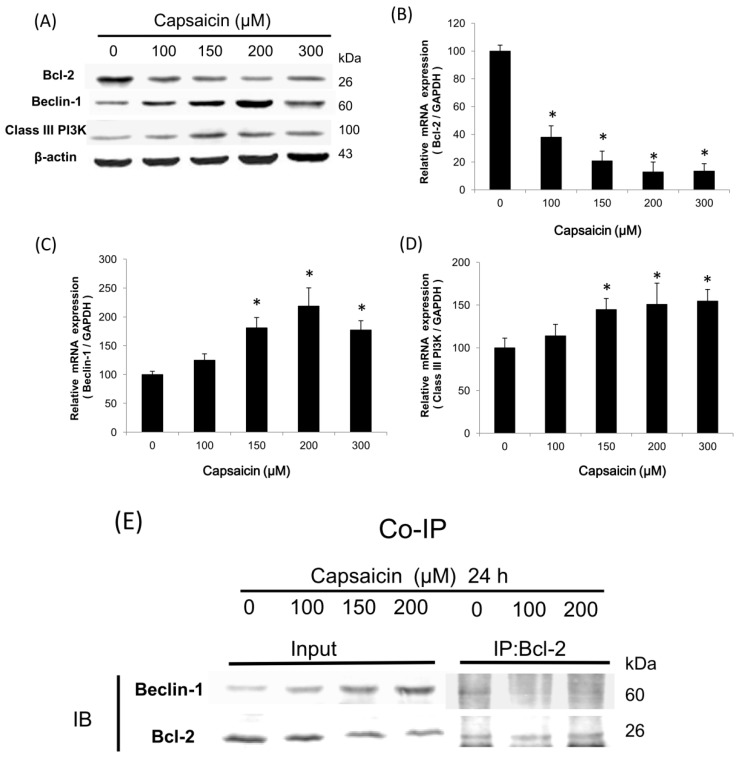
Capsaicin induces autophagy in NPC-TW01 cells. (**A**) NPC-TW01 cells were treated with capsaicin (0, 100, 150, 200 and 300 µM) for 24 h, and the protein levels of Bcl-2, Beclin-1 and class III PI3K were determined by Western blotting; (**B**–**D**) comparison of the relative mRNA expression of Bcl-2, beclin-1 and class III PI3K using qRT-PCR. Data are expressed as the percentage of the internal control (glyceraldehyde-3-phosphate dehydrogenase; GAPDH). Values were reported as the means ± SD (*n* = 6). * *p* < 0.05 vs. the control; (**E**) the interaction between Bcl-2 and Beclin-1. Bcl-2 or Beclin-1 complexes were co-immunoprecipitated from equal amounts of proteins lysates from NPC-TW01 cells treated with 0, 100 or 200 µM capsaicin for 24 h using anti-Bcl-2 antibodies. Similar results were observed in replicate experiments.

To investigate the signaling pathways involved in the induction of autophagy in capsaicin-treated NPC-TW01 cells, class III PI3K, Beclin-1 and Bcl-2 levels were examined by Western blotting and qRT-PCR. The protein levels of Beclin-1 and class III PI3K were significantly upregulated in the 150, 200 and 300 µM capsaicin-treated groups compared with the control, whereas Bcl-2 protein levels were significantly lower in the 100, 150, 200 and 300 µM capsaicin-treated groups compared with the control (Figure 3A). The mRNA expression of Bcl-2 also exhibited a downward trend in the low-dose (100 µM) capsaicin-treated groups (Figure 3B), whereas capsaicin in the 150, 200 and 300 µM active Beclin-1 and class III PI3K gene expression (Figure 3C,D). The results suggest that the class III PI3K/Beclin-1/Bcl-2 pathway could be involved in NPC cell autophagy. The interaction between Beclin-1 and Bcl-2 is thought to play a regulatory role in autophagy. As shown in Figure 3E, the activity of the Bcl-2/Beclin-1 complex was assessed by co-immunoprecipitation. The results revealed that capsaicin treatment decreased the interaction between Bcl-2 and Beclin-1. These findings suggest that capsaicin may activate autophagy in NPC cell lines by modulating the Beclin-1/Bcl-2 complex.

### 2.4. Capsaicin Upregulates LC3-II and Atg5 Expression and Downregulates p62 and Fap-1 Expression in NPC-TW01 Cells

**Figure 4:**
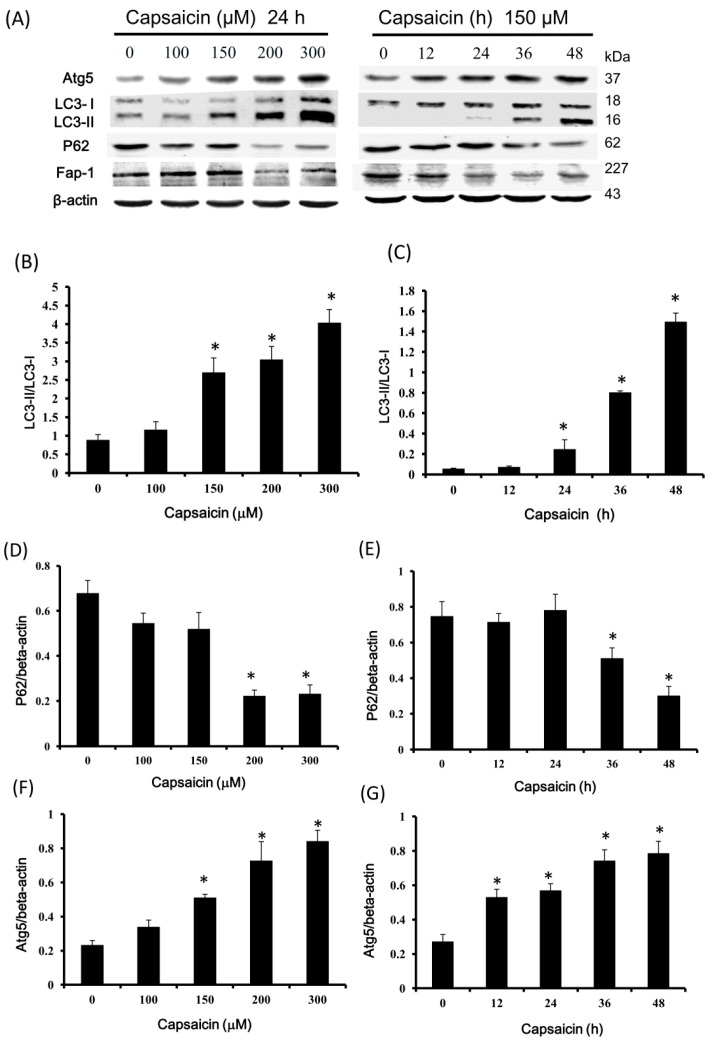

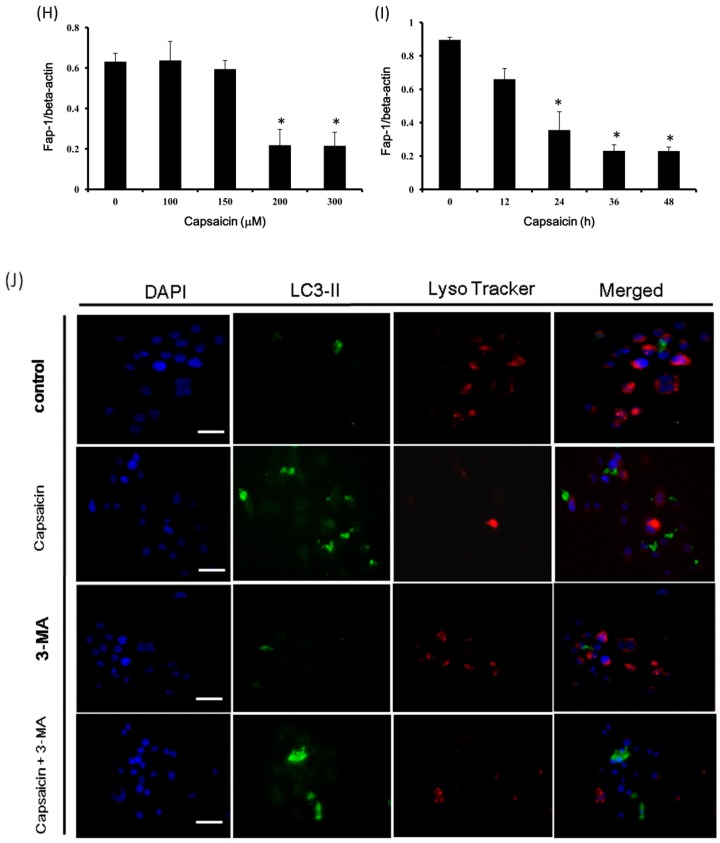
Capsaicin activates autophagy in NPC-TW01 cells. (**A**) Western blotting of Atg5, LC3, p62 and Fap-1. Cells were treated with 0, 100, 150, 200 and 300 µM capsaicin for 24 h (**left panel**) or with 150 µM capsaicin for 0, 12, 24, 36 and 48 h (**right panel**), and the expression of Atg5, LC3, p62 and Fap-1 was detected by Western blotting. Representative blots from three independent experiments are shown; (**B**–**I**): Quantification of the band intensities for Atg5, LC3, p62 and Fap-1. The results are the means ± SD of three experiments. * *p* < 0.05 vs. the control (Student’s *t*-tests); (**J**) cells were treated with vehicle (control) or 150 μM capsaicin in the presence or absence of the autophagy inhibitor 3-MA (5 mM) for 24 h. Then, LC3-II was detected by immunofluorescence staining (green). Lysosomes were labeled with LysoTracker (red), and nuclei were stained with DAPI (blue). Scale bars = 50 μm. The images shown are representative of three experiments.

Western blotting showed that the ratio of LC3-II to LC3-I and the levels of Atg5 were significantly higher in the 150, 200 and 300 μM capsaicin groups compared with the control. There was no significant difference between the 100 µM capsaicin and control groups (Figure 4A,B,F). The expression of p62 and Fap-1 was significantly lower in the 200 and 300 μM capsaicin groups than in the control group (Figure 4A,D,H). Data from the time-course study indicated that the LC3-II to LC3-I ratio was significantly higher in the 24-, 36- and 48-h capsaicin groups compared with the control, but there was no significant difference between the 12-h capsaicin and control groups (Figure 3A,C). The expression of Atg5 was significantly higher in the 12-, 24-, 36- and 48-h capsaicin groups than in the control group (Figure 4A,G). Conversely, the expression of p62 was significantly lower in the 36-h and 48-h capsaicin groups compared with the control group (Figure 4A,E). The expression of Fap-1 was significantly lower in the 24-, 36- and 48-h capsaicin groups compared with the control group (Figure 4A,I). These data suggest that autophagy was activated in NPC-TW01 cells at the elongation stage after capsaicin treatment.

In addition, LC3-II expression and subcellular distribution were assessed using LC3-II immunostaining. Cells were incubated with 150 μM capsaicin for 24 h, immunofluorescently labeled with anti-LC3-II-GFP (green) antibodies and incubated with DAPI (blue; stains the nuclei) and LysoTracker (red; an acidotropic dye that primarily detects lysosomes) (Figure 4J). LC3-II staining was more intense in capsaicin-treated compared with control cells. Furthermore, there were less LC-II-positive cells in the group treated with the autophagy inhibitor 3-methyladenine (3-MA) alone compared with the group treated with both capsaicin and 3-MA.

### 2.5. Capsaicin Induces Caspase-3 Activity in NPC-TW01 Cells

**Figure 5:**
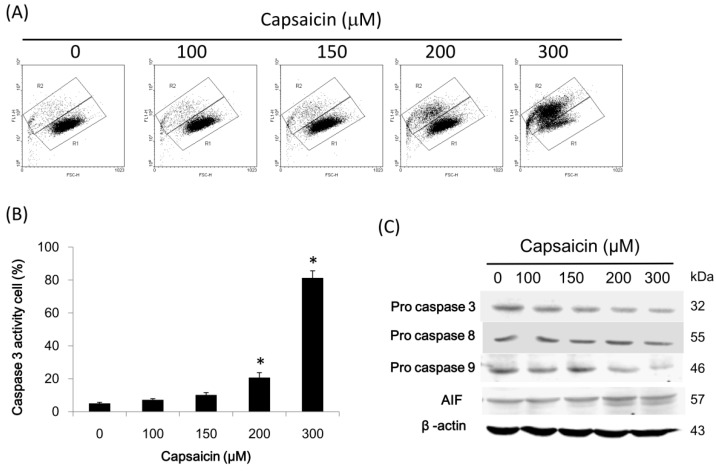
Capsaicin induces caspase cleavage and activity in NPC-TW01 cells. NPC-TW01 cells were incubated with the indicated concentrations of capsaicin for 24 h. (**A**) The cells were labeled with FITC-anti-cleaved caspase-3 antibodies, and caspase-3 activity was analyzed by flow cytometry. Cells in the *R*1 area shifted to the *R*2 area as caspase-3 activity increased in cells following capsaicin treatment; (**B**) Caspase-3 activity was quantified by measuring the percentage of cells in the R2 area. The results are reported as the means ± SD of three experiments. * *p* < 0.05 vs. control (Student’s *t*-tests); (**C**) the levels of pro-caspase-3, -8 and -9 and AIF were analyzed by Western blotting.

Next, we hypothesized that capsaicin induces NPC cells’ apoptosis by cleaving caspase-3. To explore this effect of capsaicin in NPC cells, an in vitro study was initiated by treating NPC-TW01 cells with the indicated concentrations of capsaicin for 24 h. Then, caspase-3 activity was detected by measuring FITC-labeled cleaved caspase-3 levels via flow cytometry. Data showed that cells exhibiting caspase-3 activity (R2 area) were significantly shifted from main cells (R1 area) among cells incubated with capsaicin (Figure 5A). Quantification of caspase-3 activity, measured as the percentage of cells in the R2 area, showed that caspase-3 activity was significantly increased following capsaicin treatment (Figure 5B). In addition, the levels of pro-caspase-3, -8 and -9 were measured by Western blotting. Figure 5C shows that capsaicin induced pro-caspase-3 and -9 degradation in a concentration-dependent manner. However, the pro-caspase-8 level was not affected. Taken together, the observations suggest that capsaicin significantly induced caspase-3 activity in NPC-TW01 cells.

### 2.6. Capsaicin Inhibits PI3K Expression and the Phosphorylation of Downstream Effectors of the PI3K/Akt/mTOR Pathway in NPC-TW01 Cells

**Figure 6:**
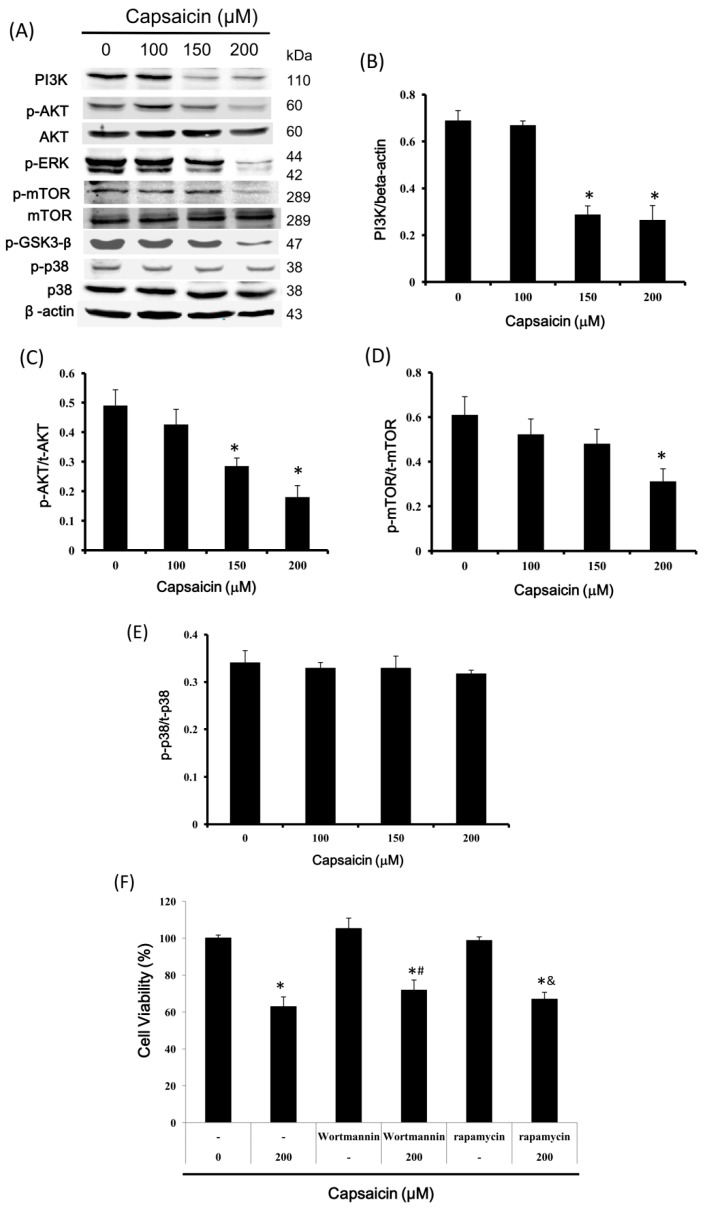
Capsaicin downregulates the PI3K/Akt/mTOR pathway members in NPC-TW01 cells. Cells were treated with capsaicin (0, 100, 150 and 200 µM) for 24 h. (**A**) The cell lysates were prepared and analyzed by Western blotting to measure PI3K, p-Akt, Akt, p-ERK, p-mTOR, p-GSK3-β, p-p38 and p38 levels; (**B**–**E**) Quantification of the band intensities of PI3K, p-Akt, p-mTOR and p-p38; (**F**) cells were treated with capsaicin (200 μM) in the presence or absence of the PI3K inhibitor wortmannin (80 nM) or the mTOR inhibitor rapamycin (2 µM) for 24 h and then analyzed by MTT assay. The results are presented as the means ± SD of three experiments. * *p* < 0.05 vs. the control; # *p* < 0.05 vs. wortmannin-only treatment; & *p* < 0.05 vs. rapamycin-only treatment (Student’s *t*-tests).

The PI3K/Akt/mTOR pathway plays an important role in regulating the cell cycle, apoptosis and autophagy. Therefore, we next investigated whether the effects of capsaicin on NPC-TW01 cells are mediated by this pathway by examining the phosphorylation of proteins in this pathway. The data showed that the phosphorylation of Akt, ERK, p-GSK3-β, and mTOR was inhibited by capsaicin treatment (Figure 6A). Capsaicin also reduced PI3K expression, suggesting that capsaicin blocks the PI3K/Akt/mTOR pathway (Figure 6B–D). However, p38 phosphorylation was not affected (Figure 6E). To clarify the effects of capsaicin and cell proliferation on the PI3K/Akt/mTOR pathway, wortmannin (PI3K inhibitor) and rapamycin (mTOR inhibitor) were used. Neither inhibitor affected the antiproliferative effects of capsaicin on NPC-TW01 cells (Figure 6F). Taken together, these data suggest that capsaicin may directly inhibit cell proliferation via the PI3K/Akt/mTOR pathway in NPC-TW01 cells.

## 3. Discussion

Capsaicin is a spicy component of hot peppers. Its chemical structure includes an aromatic ring and dipolar amide bond.

Lewinska A. et al. [31] have found that capsaicin may induce DNA and chromosomal damage in human lung (A549) and prostate (DU145) cancer cells, which may contribute to limited susceptibility of these cells to apoptotic cell death and may challenge the use of capsaicin in anticancer therapies, at least in lung and prostate cancer treatment. Capsaicin at low doses was able to stimulate both DNA double-strand breaks and micronuclei production; whereas, at concentrations of ≥100 μM, capsaicin induced a decrease in metabolic activity and cell proliferation and caused changes in the cell cycle. Capsaicin was unable to cause apoptotic cell death when used in concentrations as high as 250 μM. Capsaicin induced reactive oxygen species production, but there was no significant effect in the mitochondrial inner transmembrane potential [31]. Also worth noting, capsaicin with 200 to 300 μM was found to induce ladder-shaped nucleosomal DNA fragments in human pharyngeal squamous carcinoma (FaDu) cells [23]; at 300 μM, capsaicin induced apoptosis in NPC-TW039 cells [24].

In this study, our data showed that capsaicin induced reactive oxygen species generation when NPC cells were treated with increasing doses of capsaicin, but was ineffective in causing the disruption of the mitochondrial transmembrane potential (ΔΨm) (data not shown). In addition, we found that capsaicin at 100 μM induced autophagy via the activation of the class III PI3K/Beclin-1/Bcl-2 signaling pathway (Figure 3); at 150 μM, capsaicin caused autophagosome formations because of the increase in Atg and LC3-ll levels (Figure 4), and capsaicin at 300 μM triggered NPC-TW01 cell apoptosis due to increases in caspase-3 activity (Figure 5); thereby confirming that capsaicin stimulated NPC cells’ autophagy at lower concentrations.

Beclin-1, a novel BH3-only protein, is a key component of the class III PI3K complex, which is involved in the initiation of autophagosome formation. It was recently reported that Beclin-1 interacts with Bcl-2 and Bcl-xL to inhibit autophagy [32,33,34,35,36]. The current results indicated that capsaicin treatment increased the protein levels of Beclin-1 and class III PI3K and decreased the levels of Bcl-2. Capsaicin also affected the interaction between Bcl-2 and Beclin-1. Therefore, capsaicin may activate autophagy in NPC cells via the class III PI3K/Beclin-1/Bcl-2 pathway.

Cell cycle progression involves the sequential activation of CDKs, which requires association with corresponding regulatory cyclins for activation [37,38]. For example, the G1-S-phase transition is regulated by complexes formed by cyclin D and CDK4 or CDK6, cyclin E and CDK2 [37,38]. In the current study, we determined the effects of capsaicin treatment on the levels of G1-S-specific cyclins and CDKs by Western blotting to gain insight into the mechanism behind capsaicin-induced cell cycle arrest in NPC. The results revealed that capsaicin induced cell cycle arrest in G1 phase and reduced the protein levels of cyclin D1, CDK4, cyclin E and CDK2 in TW-01 NPC cells. Therefore, capsaicin-induced G1 phase arrest in NPC cells could be attributed to the downregulation of G1-S-specific cyclins and CDKs.

Atg5 forms a conjugate with Atg12 to play a key role in autophagosome formation. The LC3-II to LC3-I ratio was reported to be proportional to the number of autophagic vacuoles [39]. P62 is a polyubiquitin-binding protein that contains an LC3-interacting motif and a ubiquitin-binding domain. By linking ubiquitinated substrates to the autophagic machinery, p62 is incorporated into and degraded in autolysosomes, together with its bound proteins [40]. The current data indicate that the levels of the autophagy markers LC3-II and Atg5 were increased, whereas the level of p62 was decreased by capsaicin treatment. In addition, GFP-LC3-II-labeled fluorescence microscopy was used to confirm autophagosome formation in capsaicin-treated cells. These results suggest that capsaicin may induce autophagy in NPC cells. We also investigated apoptosis- and autophagy-relevant signaling pathways. Recent reports showed that autophagic degradation of Fap-1 promotes Fas-induced apoptosis [41,42]. The autophagy adaptor protein p62 (also known as SQSTM1) directly inter-acts with Fap-1 to recruit it to autophagosomes for degradation [41]. Under high autophagic flux, more Fap-1 is targeted for autophagy-mediated degradation, and Fas receptors remain phosphorylated and are expressed in abundance on the cell membrane. This allows more Fas signaling, which increases the activation of the extrinsic apoptotic pathway and consequently apoptosis [41]. Here, we demonstrated that both p62 and Fap-1 levels were decreased following capsaicin treatment. In addition, the current data suggest that decreased levels of Bcl-2 induced by a low capsaicin concentration (100 μM) and increased caspase-3 activity induced by a high capsaicin concentration (300 μM) triggered NPC cell apoptosis.

Finally, we investigated the mechanism potentially involved in the abovementioned effects of capsaicin in NPC cells. Akt promotes cyclin D1 translation by indirectly activating mTOR. Conversely, mTOR increases the translation of cyclin D1 by activating the ribosomal protein S6K and inhibiting eukaryotic translation initiation factor 4E-binding protein, thus increasing eIF4e activity [43,44]. PI3K/Akt signaling plays a key role in cell survival, and capsaicin may inhibit Akt phosphorylation. As a downstream effector of Akt, mTOR suppresses autophagy [45,46]. Similar concentrations of capsaicin inhibited the phosphorylation of mTOR and induced autophagy in NPC-TW01 cells, suggesting that the effects of capsaicin on inducing autophagy might be exerted by inhibiting the Akt/mTOR pathway. Cell senescence is a state of stable, irreversible proliferation arrest that is characterized by a large and flattened morphology and elevated SA-β-gal activity [47]. Although we also measured cell senescence in capsaicin-treated NPC cells, no cell senescence was detected in the current study (data not shown).

Dihydrocapsaicin (DHC), an analog of capsaicin, induces autophagy in human colon cancer cells and regulated p53 status in breast cancer cells [48]. Lewinska A. et al. in 2015 [49] indicate that a capsaicin analogue, namely capsaicin epoxide, is a potential bioactive component for anti-proliferative activity in vitro, when used at much lower concentrations compared with capsaicin at similar concentrations. Therefore, to become a drug candidate, capsaicin must exert favorable in vivo antitumor activity of NPC, which we will investigate in our next study.

## 4. Materials and Methods

### 4.1. Materials

3-(4,5-Dimethylthiazol-2-yl)-2,5-diphenyltetrazolium bromide (MTT) and dimethyl sulfoxide (DMSO) were purchased from Sigma-Aldrich (St. Louis, MO, USA). Dulbecco’s Modified Eagle′s Medium (DMEM), fetal bovine serum (FBS), phosphate-buffered saline (PBS), sodium pyruvate, trypsin and antibiotics were purchased from Gibco, BRL (Grand Island, NY, USA). The specific inhibitors 3-methyladenine (3-MA), wortmannin and rapamycin were purchased from Merck Millipore (Merck KGaA, Darmstadt, Germany). All reagents and compounds were of analytical grade.

### 4.2. Cells

NPC-TW01, which is a nasopharyngeal carcinoma (NPC) cell line that was provided by Chin-Tarng Lin’s laboratory (National Taiwan University, Taipei, Taiwan) and maintained in DMEM supplemented with 10% FBS, 2 mM l-glutamine, 100 U/mL penicillin and 100 mg/mL streptomycin in 10-cm culture dishes at 37 °C under a humidified atmosphere with 5% CO_2_ and 95% air.

### 4.3. Cell Proliferation Assays

Cells were seeded in 96-well culture plates at a density of 5000/well. The cells were exposed to 0, 50, 100, 150, 200 and 300 µM capsaicin for 24 to 48 h and then treated with MTT (1 mg/mL) for at least 4 h. The reaction was stopped by adding DMSO, and the optical density at 540 nm was measured using a multi-well plate reader. The background absorbance of medium in the absence of cells was subtracted from the absorbance readings of the reaction wells. All samples were assayed in at least triplicate, and the means were calculated for each experiment. The results are expressed as a percentage of the control, which was considered to be 100%. All assay results are expressed as the means ± SEM.

### 4.4. Cell Cycle Distribution Analysis

For cell cycle analysis, the fluorescent nucleic acid dye propidium iodide (PI) was used to identify the proportions of cells in each of the three interphases of the cell cycle. Cells were treated with capsaicin, harvested and fixed in 1 mL cold 70% ethanol for at least 8 h at −20 °C. DNA was then stained in PI/RNaseA solution, and the cell cycle (at least 10,000 single cells) was analyzed using a FACSCalibur flow cytometer (Becton-Dickinson, San Jose, CA, USA). Data were analyzed using WinMDI 2.9 software (Becton-Dickinson, San Jose, CA, USA).

### 4.5. Quantitative Real-Time Reverse Transcriptase PCR 

Total RNA was extracted using TRIzol reagent (Invitrogen, Carlsbad, CA, USA). Total RNA (5 μg) was reverse transcribed, and 1 μg of the RT product was subjected to PCR in the presence of specific primers. The sequences of the primers were as follows: Bcl-2: 5′-GCCACTTACCTGAATGACCACC-3′ and 5′-AACCAGCGGTTGAAGCGTTCCT-3′; beclin-1: 5′-GAGGGATGGAAGGGTCTAAG-3′ and 5′-GCCTGGGCTGTGGTAAGT-3′; class III PI3K: 5′-CTCACCAAAGCTCATCGACA-3′ and 5′-CATCGAAATTCAACCATCAGG-3′; and GAPDH (glyceraldehyde-3-phosphate dehydrogenase): 5′-GTCTCCTCTGACTTCAACAGCG-3′ and 5′-ACCACCCTGTTGCTGTAGCCAA-3′. qRT-PCR was performed on the ABI 7300 system (Applied Biosystems, Foster City, CA, USA) in 30-μL reactions using the following program: 40 cycles of 95 °C for 120 s, 60 °C for 30 s and 72 °C for 30 s. All samples were amplified in triplicate in one assay run simultaneously. GAPDH was included in each reaction as the internal standard, and relative gene expression was quantified using the 2^−ΔΔ*C*t^ method. Values are expressed as the percentage of the internal control (GAPDH).

### 4.6. Western Blotting

Cells were lysed in RIPA buffer, and the protein contents were quantified using the BCA Protein Assay Kit (Thermo, Waltham, MA, USA). Briefly, the proteins were separated by SDS-PAGE and then transferred to PVDF membranes (Millipore). After blocking with 5% nonfat milk, the membranes were blocked with blocking buffer (Odyssey; LI-COR, Lincoln, NE, USA) overnight. The membranes were then incubated with primary antibodies against the following proteins for 90 to 120 min: β-actin (Sigma-Aldrich; loading control), LC3 (Sigma-Aldrich), P62 (Proteintech, Chicago, IL, USA), Atg5 (N18; sc-8666; Santa Cruz Biotechnology, Santa Cruz, CA, USA), Bcl-2 (N-19; sc-492), Beclin-1 (H-300, sc-11427), PI3KC3 (AP8014a; Abgent, San Diego, CA, USA), cyclin D1 (H-295; sc-753), cyclin E (M-20; sc-481), cyclin-dependent kinase (CDK) 4; H-22; sc-601), CDK2 (H-298; sc-748), CDK6 (C-21; sc-177), pro-caspase-3 (31A1067; sc-56053), pro-caspase-8 (T16; sc-61334), pro-caspase-9 (H-83; sc-7885), apoptosis inducing factor (AIF; E-1; sc-13116), PI3K-p100 (H-300; sc-134986), p-mTOR (Ser-2448; sc-101738), p-GSK3-β (H-79; sc-9166), p-Akt (Ser-473; sc-7985-R), Akt (H-136; sc-8312), p-ERK (E-4; sc-7383), p38 (A-12; sc-7972), p-p38 (D-8; sc-7973) and Fap-1 (NB100-56139; Novus, Littleton, CO, USA). The membranes were then incubated with the appropriate horseradish peroxidase-conjugated second antibodies (diluted 1:20,000) (IRDy; LI-COR) 30 to 45 min. Next, the antigens were visualized on a near-infrared imaging system (Odyssey; LI-COR) and data were analyzed using the software Odyssey 2.1 software (Odyssey; LI-COR).

### 4.7. Co-Immunoprecipitation 

Co-IP is an effective approach for quantifying protein–protein interactions in cells. Briefly, after incubation at room temperature overnight, 500 mg cellular protein were labeled with anti-Bcl-2 antibodies (N19; sc-492; Santa Cruz Biotechnology, Santa Cruz, CA, USA). The protein-antibody complexes were then collected using protein A/G plus agarose beads (sc-2003; Santa Cruz Biotechnology). Following the final wash, the samples were boiled and centrifuged to pellet the agarose beads. The immunoprecipitates were analyzed by SDS-PAGE followed by Western blotting using anti-Beclin-1 or -Bcl-2 antibodies. Western blotting was performed to analyze proteins in the supernatant, and antigens were visualized using a chemiluminescence detection kit (Amersham Corp., Arlington Heights, IL, USA). The data were analyzed using Odyssey 2.1 software (Odyssey; LI-COR).

### 4.8. Immunofluorescence Staining

Cytosolic 18 kDa LC3-I is converted into autophagosome-associated 16-kDa LC3-II via conjugation with phosphatidylethanolamine. This conversion is a reliable marker of autophagy [25]. Cells plated on coverslips were fixed in 4% paraformaldehyde. After three washes with PBS, the cells were permeabilized with 0.3% Triton X-100 for 5 min and then incubated in blocking solution at room temperature for 1 h, followed by anti-LC3B primary antibodies (L7543; Sigma-Aldrich, St. Louis, MO, USA) for 1 h. Next, the cells were incubated with Alexa Fluor-labeled secondary antibodies (diluted 1:500) for 1 h and washed with PBS. The coverslips were mounted in Prolong Gold anti-fade reagent with 4′,6-diamidino-2-phenylindole (DAPI; Carlsbad, CA, USA) for 2 min and inspected under a confocal microscope (CARV II, Becton-Dickinson, San Jose, CA, USA).

### 4.9. Senescence-Associated β-Galactosidase Staining

Cell senescence was detected using the Senescence β-Galactosidase Staining Kit (No. 9860, Cell Signaling Technology, Inc., Danvers, MA, USA) and phase contrast microscopy (Olympus CKX41; Tokyo, Japan) according to the manufacturer’s instructions. Cells were seeded in 24-well culture plates at a density of 1.5 × 10^5^/well. After treatment with capsaicin, the culture media were removed, and the cells were washed once with 1 mL PBS. The cells were then fixed with 0.5 mL fixative solution at room temperature for 15 min. The cells were rinsed twice with 1 mL PBS and then incubated with the staining mixture (470 μL staining solution, 5 μL staining supplement and 25 μL 20 mg/mL X-gal in DMF (*N*,*N*-dimethylformamide)) at 37 °C without CO_2_ overnight. The cells were observed under a microscope for the development of a blue color.

### 4.10. Caspase-3 Activity Assay

Caspase-3 activity was detected using the Caspase-3 (active) FITC Staining Kit (ab65613). After treatment with capsaicin, the cells (1 × 10^6^/mL) were collected by centrifugation and washed with PBS. Next, 1 μL FITC-anti-caspase-3 antibody was added to each tube and incubated for 30 min at 37 °C in an incubator with 5% CO_2_. The supernatant was removed by centrifugation at 3000 rpm for 5 min, and the cells were resuspended in 0.5 mL wash buffer and centrifuged again. Finally, the stained cells were analyzed using the FACSCalibur flow cytometer (Becton-Dickinson, San Jose, CA, USA). An additional negative control was prepared by adding 1 μL/mL of the caspase inhibitor Z-VAD-FMK to an induced culture to inhibit caspase-3 activation. The data were analyzed using WinMDI 2.9.

### 4.11. Statistical Analysis

Data are presented as the means ± SD. Statistical comparisons between two groups were made using unpaired Student’s *t*-tests. Differences among groups were tested using one-way analysis of variance with Tukey’s post hoc tests. In all cases, differences were considered statistically significant when *p* < 0.05.

## 5. Conclusions

In conclusion, capsaicin exerts potent effects to induce G1 arrest and autophagy in NPC-TW01 cells. These effects might involve increasing class III PI3K expression by modulating the interaction between Bcl-2 and beclin-1. Capsaicin treatment induced cell autophagy by increasing LC3-II and Atg5 levels, decreasing p62 and Fap-1 expression and increasing caspase-3 activity to induce apoptosis in human NPC cells. This suggests that there is a correlation between the downregulation of PI3K expression and the phosphorylation of downstream effectors in the PI3K/Akt/mTOR pathway. Capsaicin may become a promising drug candidate for NPC cancer therapy.

## Abbreviations

NPC	Nasopharyngeal carcinoma
STATs	Signal transducer and activator of transcription protein family
GAPDH	Glyceraldehyde-3-phosphate dehydrogenase
MTT	3-(4,5-Dimethylthiazol-2-yl)-2,5-diphenyltetrazolium bromide
DMSO	Dimethyl sulfoxide
DMEM	Dulbecco’s Modified Eagle’s Medium
FBS	Fetal bovine serum
PBS	Phosphate-buffered saline
PVDF	Polyvinylidene fluoride
3-MA	3-methyladenine
3-methyladenine	Propidium iodide
CDK	Cyclin-dependent kinase
Atg5	Autophagy protein 5
AIF	Apoptosis inducing factor
qRT-PCR	Quantitative real-time reverse transcription polymerase chain reaction
Co-IP	Co-immunoprecipitation
DAPI	4′,6-diamidino-2-phenylindole
DMF	*N*,*N*-dimethylformamide
